# Supplementary material for: Glioblastoma Cells Use an Integrin- and CD44-Mediated Motor-Clutch Mode of Migration in Brain Tissue
Source: Cell Mol Bioeng. 2024 Mar 4;17(2):121–35. doi: 10.1007/s12195-024-00799-x (PMC11082118; doi:10.1007/s12195-024-00799-x)
Supplement: Supplementary file 7 — Supplementary file7 (DOCX 1172 kb) [file 12195_2024_799_MOESM7_ESM.docx]

**Supplementary Figures**


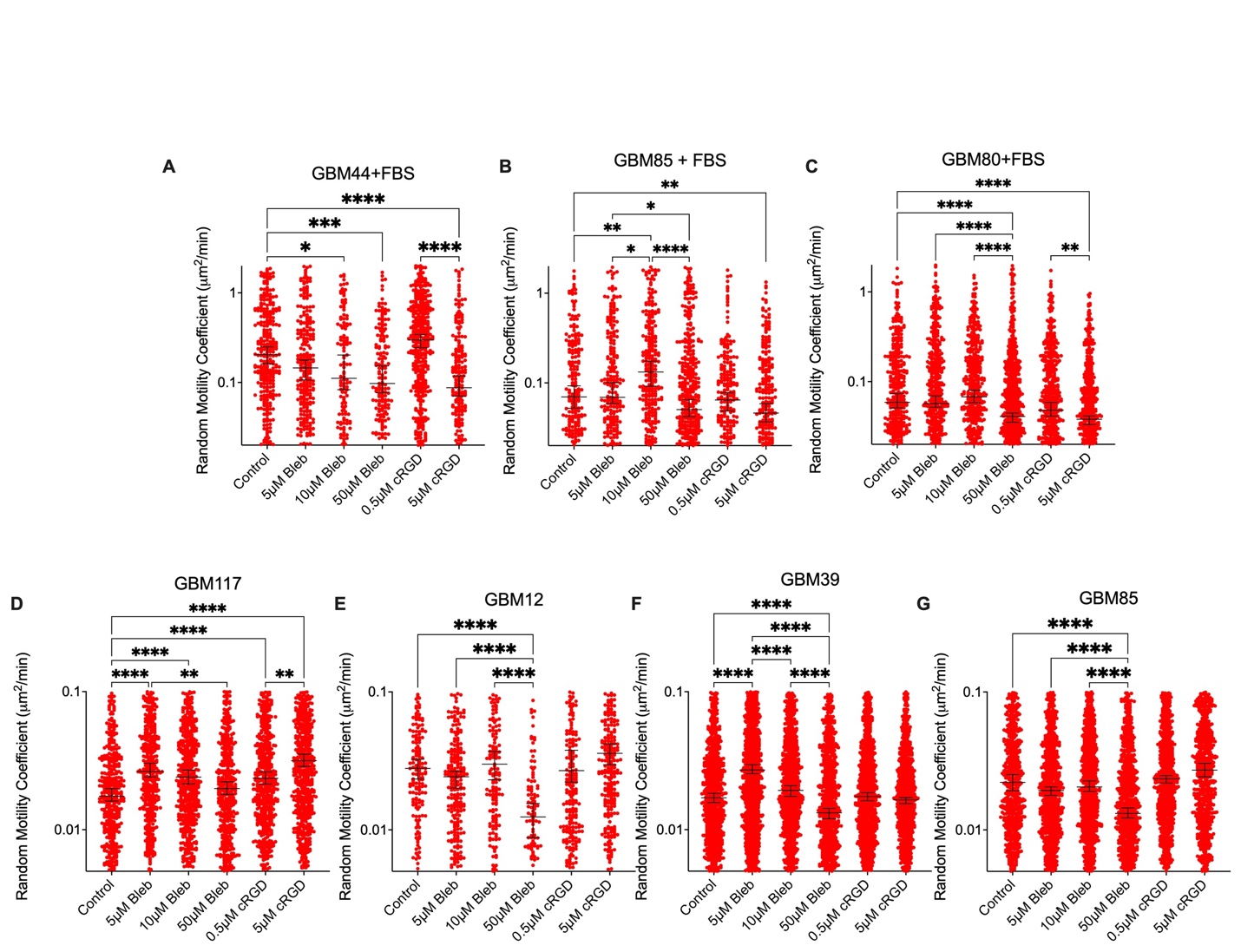


Supplementary Figure 1. Migratory behavior of individual glioma cells in brain slices in response to blebbistatin and cyclo-RGD. RMC of cells in response to varying doses of blebbistatin and cRGD for A) GBM44 + FBS, B) GBM 85 + FBS, C) GBM 80 + FBS, D) GBM 117, E) GBM 12, F) GBM 39, and G) GBM 85. Each dot represents a single cell. The black line denotes the median value and 95% confidence interval. Statistics were done using a Kruskal Wallis test.


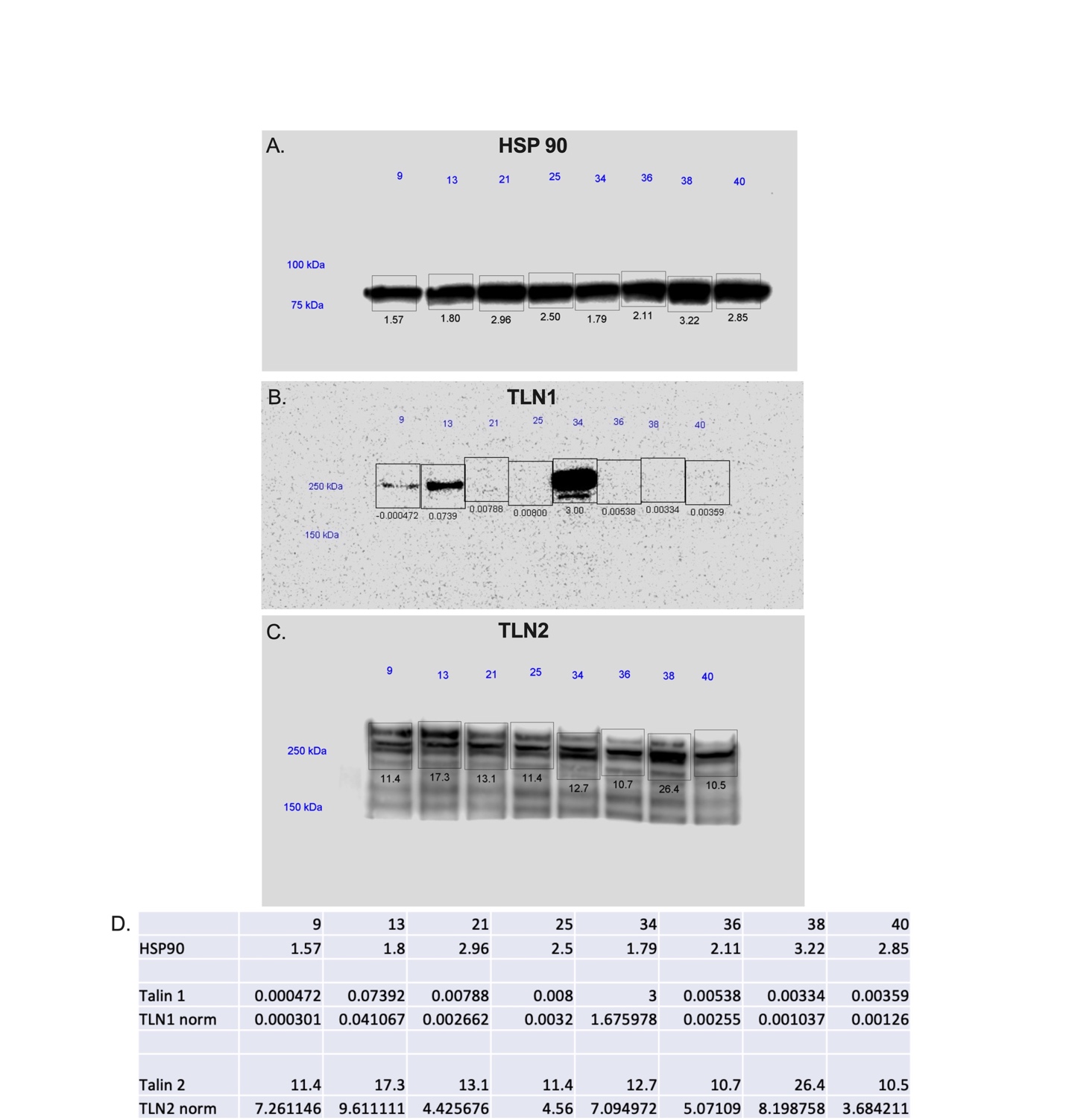


Supplementary Figure 2. Western Blot demonstrating knockout of talin 1. Western Blot results for the A) control HSP90, B) Talin1, and C) Talin2 in development of Talin1 knockout. D) Calculations demonstrating knockout of talin1 in given samples and no significant upregulation of talin 2.


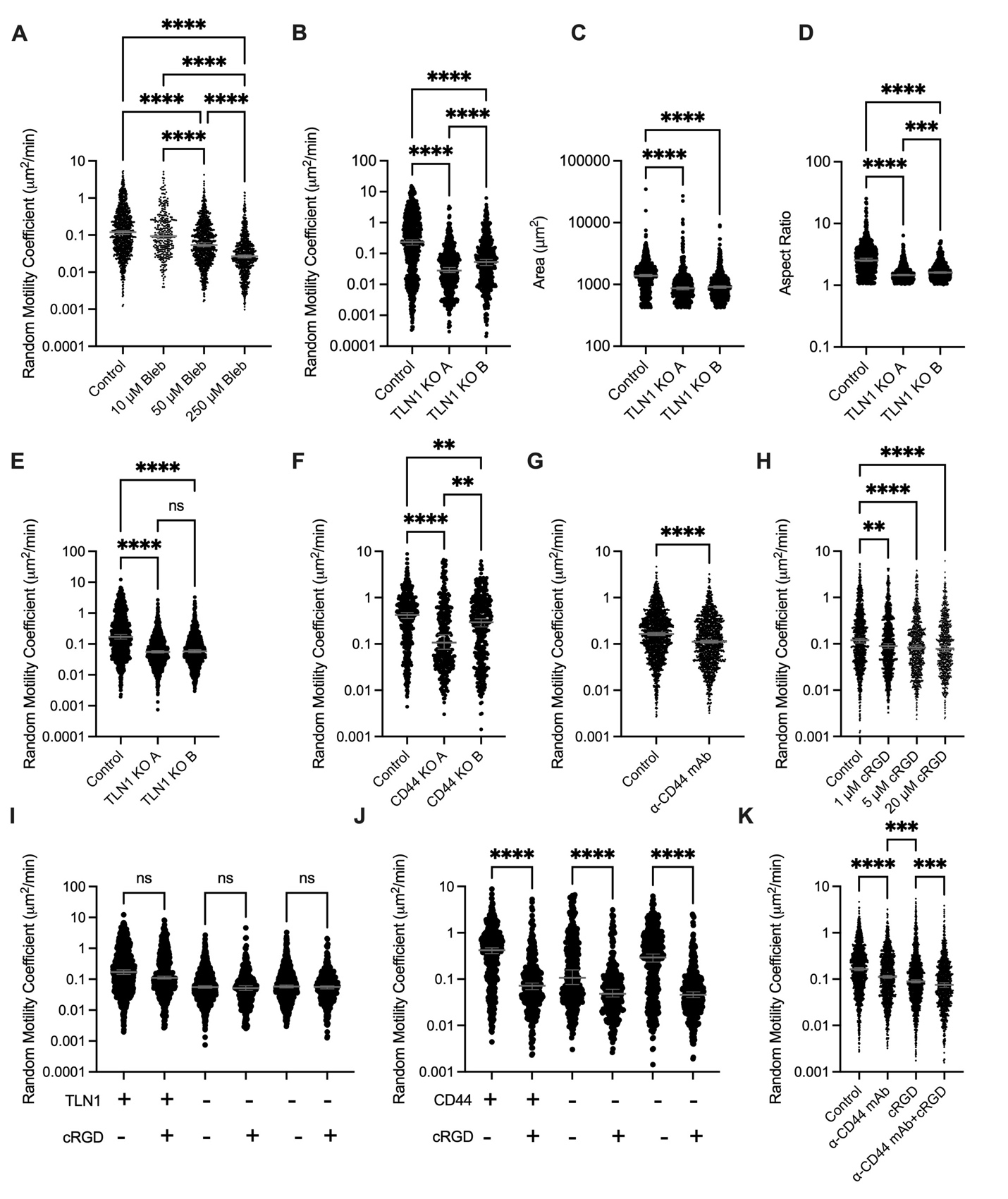
Supplementary Figure 3. Scatter plots of migration experiments showing individual data points. The data is pooled from all 3 trials and each dot represents a single cell. A) RMC of U251 cells in response blebbistatin. B) RMC, C) Area, and D) Aspect Ratio of TLN1 KO cells on collagen PAGs. E) RMC of TLN1 knockout cells in brain tissue. F) RMC of CD44 KO cells in brain tissue. G) RMC of U251 cells in response to α-CD44 mAb. H) RMC of U251 cells in response to varying doses of cyclo-RGD. I) RMC of U251 cells +/- TLN1 KO in response to cyclo-RGD. J) RMC of U251 cells +/- CD44 KO in response to cyclo-RGD. K) RMC of U251 cells in response to cyclo-RGD and α-CD44 mAb.

**Supplementary Movie Captions**

Supplementary Movie 1. PDX cell pulls on vasculature. Example movie of GBM44 (green) pulling on vasculature (magenta) as it migrates.

Supplementary Movie 2. PDX cell pulls on vasculature. Example movie of GBM44 (green) pulling on vasculature (magenta) as it migrates.

Supplementary Movie 3. U251 cell pulls on vasculature. Example movie of U251 (green) pulling on vasculature (magenta) as it migrates.

Supplementary Movie 4. U251 cell pulls on vasculature Example movie of U251 (green) pulling on vasculature (magenta) as it migrates.

Supplementary Movie 5. U251 cells migrating in brain tissue. Example movie of U251 cells (green) migrating in brain tissue with vasculature and microglia (magenta).

Supplementary Movie 6. PDX cells migrating in brain tissue. Example movie of GBM80 cells (green) migrating in brain tissue with vasculature and microglia (magenta).
